# Governing Personalized Health: A Scoping Review

**DOI:** 10.3389/fgene.2021.650504

**Published:** 2021-04-21

**Authors:** Philipp Trein, Joël Wagner

**Affiliations:** ^1^Department of Political Science and International Relations, University of Geneva, Geneva, Switzerland; ^2^Department of Actuarial Science, Faculty of Business and Economics (HEC Lausanne), University of Lausanne, Lausanne, Switzerland; ^3^Swiss Finance Institute, University of Lausanne, Lausanne, Switzerland

**Keywords:** personalized health, research infrastructure, trust, regulation, health system, governance, policymaking

## Abstract

Genetic research is advancing rapidly. One important area for the application of the results from this work is personalized health. These are treatments and preventive interventions tailored to the genetic profile of specific groups or individuals. The inclusion of personalized health in existing health systems is a challenge for policymakers. In this article, we present the results of a thematic scoping review of the literature dealing with governance and policy of personalized health. Our analysis points to four governance challenges that decisionmakers face against the background of personalized health. First, researchers have highlighted the need to further extend and harmonize existing research infrastructures in order to combine different types of genetic data. Second, decisionmakers face the challenge to create trust in personalized health applications, such as genetic tests. Third, scholars have pointed to the importance of the regulation of data production and sharing to avoid discrimination of disadvantaged groups and to facilitate collaboration. Fourth, researchers have discussed the challenge to integrate personalized health into regulatory-, financing-, and service provision structures of existing health systems. Our findings summarize existing research and help to guide further policymaking and research in the field of personalized health governance.

## Introduction

In the wake of the Human Genome Project and the development of new digital technologies, a whole range of novel possibilities emerges for public health and health care. In referring to labels such as personalized health, precision medicine, personalized medicine, stratified health, or 4P (predictive, preventive, personalized, and participatory) medicine (Chataway et al., 2012; Flores et al., 2013; Andreu-Perez et al., 2015; Heart et al., 2017), researchers have explored how technological innovations for preventing and treating diseases can be translated into health practice (Zeggini et al., 2019). In the following, we refer to the ensemble of these terms as personalized health (PH).

Against this background, some scholars and practitioners have raised high hopes regarding the potential of integrating PH in existing health systems (Chambers et al., 2016), whereas others have cautioned against too high expectations (Joyner and Paneth, 2015; Snyderman et al., 2016). Regardless of how PH will affect the creation of value in the practice of health care and public health, translating PH into the governance of health is an important problem and a challenge for policymakers (Stark et al., 2019) as well as a political problem. It requires regulatory efforts, such as frameworks protecting patients and citizens against discrimination based on their genetic profiles (Green et al., 2015). In addition, there is a demand for regulation and the creation of incentives for providers and payers to develop products for medical markets (Phillips et al., 2014; Kukk et al., 2016). Whilst many publications have pointed to some political challenges regarding the implementation of personalized health, a comprehensive summary of the most important governance issues regarding personalized health is lacking.

In this article, we contribute to the literature, in mapping of the research dealing with PH governance in a wider sense. Governance refers to the process and the results of the coordination of public and private actors involved in (public) policymaking (Kickbusch and Gleicher, 2012) at the national and global level (Frenk and Moon, 2013). We conduct a scoping review, which focuses on the governance challenges that policymakers (defined in a wide sense, including various private, and public stakeholders) face in order to implement a more personalized approach to health care. Therefore, we map four related challenges, which policymakers face when putting research into practice to create economic and social value from PH (Florin and Escher, 2017).

Our analysis points to four governance challenges that decisionmakers face against the background of personalized health. First, researchers have highlighted the need to further extend and harmonize existing research infrastructures in order to combine different types of genetic data. Second, decisionmakers face the challenge to create trust in personalized health applications, such as genetic tests. Third, scholars have pointed to the importance of the regulation of data production and sharing to avoid discrimination of disadvantaged groups and to facilitate collaboration. Fourth, researchers have discussed the challenge to integrate personalized health into regulatory-, financing-, and service provision structures of existing health systems. Our findings summarize existing research and help to guide further policymaking and research in the field of personalized health governance.

## Review Strategy and Method

We conduct a scoping review of the literature, using the procedure for this method described in the literature (Arksey and O’Malley, 2005; Thomas et al., 2017). Such an approach is suitable as our goal is to broadly map existing research dealing with governance and public policy of PH, rather than to analyze more specific research questions. We base our review on a search in the Web of Science Core Collection database (cf. webofknowledge.com). We include all years (1900–2019), all document types (journal articles, books, chapters, conference proceedings, etc.), as well as all Web of Science categories, i.e., subject areas. We decided to focus our search on one comprehensive database to keep our review feasible.

**FIGURE 1:**
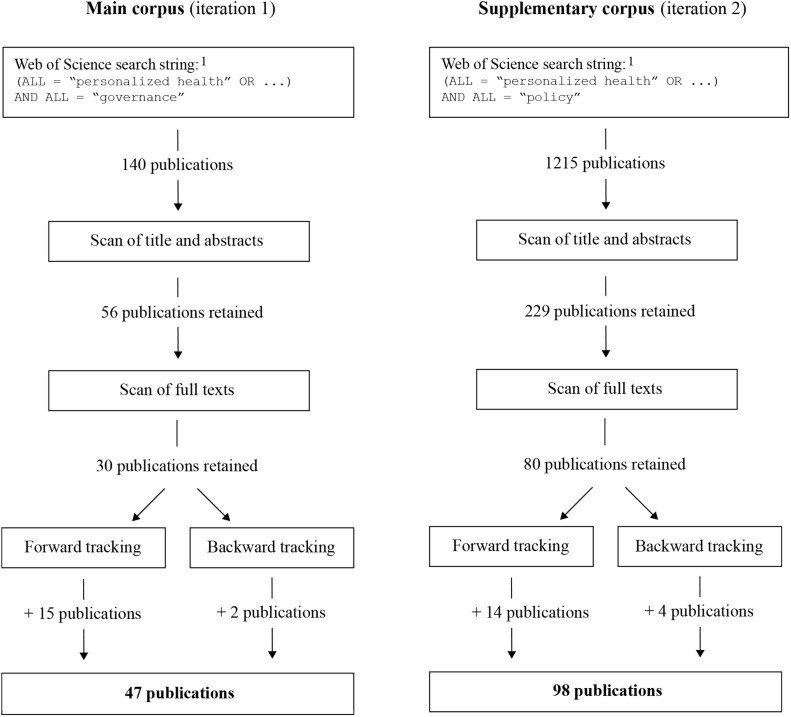
Review protocol.

Since our review focuses on (latent) theoretical concepts, i.e., governance and public policy, we search the literature in two iterations (see Figure 1). The first search step combines different terms related to PH with “governance.” In the second search step, we replace governance with “policy.” To operationalize PH in the search, we use the following search terms: “personalized health,” “personalized health care,” “precision medicine,” “individualized medicine,” “personalized medicine,” “stratified medicine,” “genetic medicine,” and “genomic medicine.” We also include all combinations of alternative spellings (personalized/personalised, individualized/individualised, health care/healthcare). The exact search string for the first iteration is (ALL = “personalized health” OR ALL = “personalised health” OR ALL = “personalized healthcare” OR ALL = “personalised healthcare” OR ALL = “personalized health care” OR ALL = “personalised health care” OR ALL = “precision medicine” OR ALL = “individualized medicine” OR ALL = “individualised medicine” OR ALL = “personalized medicine” OR ALL = “personalised medicine” OR ALL = “stratified medicine” OR ALL = “genetic medicine” OR ALL = “genomic medicine”) AND ALL = “governance.” For the second iteration, the chain of keywords is the same but we replace “governance” with “policy” at the end of the search string.

We define governance essentially as the process and result of public and private stakeholder coordination with the goal to actively solve problems through public policy (Goetz, 2008; Trein et al., 2019). The term governance does also include stakeholders beyond elected officials and bureaucrats. Therefore, we focus on “policy” as an alternative search term for governance, rather than “legislation” or “law.” This approach ensures that we do not miss articles that are substantially interesting to us but do not use the term governance.

To conduct the literature review, we followed a five-step process (Figure 1). Firstly, we carried out the search in the Web of Science database using the above-mentioned keywords. Secondly, we read the title and abstract of the publications to verify if they are interesting for our topic and excluded those that do not fit. Thirdly, we scanned the full-text of the publications and retained those that corresponded to the criteria we are interested in. Fourth, we pursued a for-ward tracking search of the articles, i.e., we skimmed the titles of the publications that cite the selected works in Google Scholar (cf. scholar.google.com). Finally, we embarked in backward tracking, which entailed scanning the bibliography of the selected publications.

Our search results in two corpora of publications (Figure 1). The main corpus contains those publications that have emerged from the selection procedure of the first iteration (focusing on governance). The publications resulting from the second iteration (policy) form the corpus of supplementary publications. The sizes of both corpora differ, which is due to the two different populations from which we started our search but also as authors use the term policy much more frequently than governance. The publications that we retained for the main corpus are journal articles, one book and one book chapter. The supplementary material corpus contains mostly articles as well as four books and two book chapters.

The scanning of titles, abstracts, full-texts, backward and forward tracking, as well as the subsequent reading applied two themes covering governance of PH as inclusion and exclusion criteria for the review process. We developed these themes based on the literatures related to governance (Ansell and Torfing, 2016), public policy (Knoepfel et al., 2011), health policy (Papanicolas et al., 2013; Robert et al., 2017; Reibling et al., 2019), and health governance research (Böhm et al., 2013). Specifically, the papers that corresponded to the following criteria were included and retained in the corpus throughout the different search iterations:

1. Regulations, e.g., laws, guidelines and voluntary codes, regarding PH and individualized medicine provision, for example regarding quality standards for precision medicine. This includes but is not restricted to regulations of financing and providing preventative and curative interventions as well as on research and data-related infrastructure.
2. Public and private actors involved in (public) policymaking regarding PH and individualized medicine, such as administrations, governments, health insurers, doctors, pharmaceutical agencies, patient organizations, and the relations between them, as well as conflicts and coordination between these actors.

The first author conducted most of the scoping review and selected the papers. The second author re-selected some of the papers as a validity test. The substantial analysis of the publications’ content uses thematic analysis, which is appropriate for the procedure of a scoping review (Thomas et al., 2017). For each of the 47 publication in the main corpus, we record the region that the paper covers, the methodology the authors use, the key contents and main results, as well as to which of the main challenges for the governance of PH the paper relates to (cf. the synopsis of the reviewed papers provided in the appendix). In addition, we use the publications from the supplementary corpus of papers (98) to complement the discussion of the findings. The supplementary corpus of papers were selected according to the logic as the main corpus but they were not thematically analyzed according to the themes generated through the inductive analysis of the first corpus. We use the second corpus as a robustness check by picking articles for additional examples to the narrative presented in the review. As we manage to identify papers in the control group that corresponded to the themes in the main corpus, we conclude that the governance challenges we identify in the following are valid and can be measured in another selection of papers.

## Four Challenges for PH Governance

**FIGURE 2:**
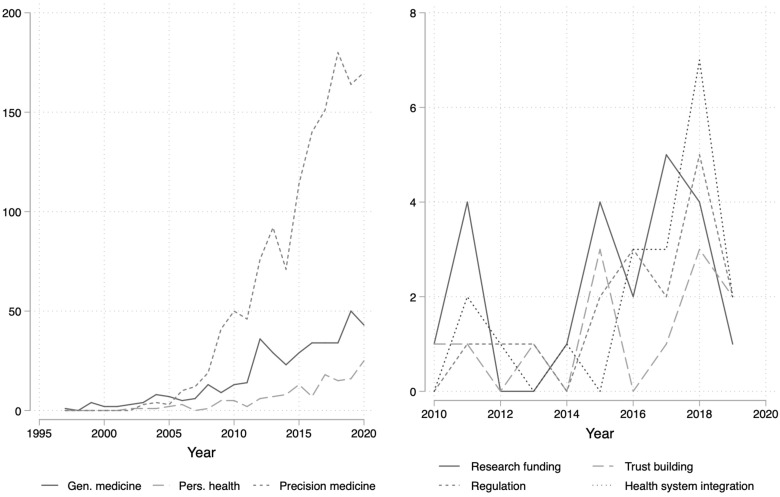
Development of publications over time.

To present the results from our literature review, we start with a descriptive overview of the publications, focusing at the overall results from our Web of Science search (left graph, Figure 2) as well as the specific findings from the thematic analysis of the main corpus (right graph, Figure 2). In order to illustrate the overall search results, we comprised three groups of search terms that we used for the review. The first group is entitled *genetic medicine* and comprises of “genetic medicine” or “genomic medicine.” The second group is named *personalized health* and contains the terms “personalized health,” “personalised health,” “personalized healthcare,” “personalised healthcare,” “personalized health care,” or “personalised health care.” The third group focuses on *precision medicine* and contains publications labeled with “precision medicine,” “individualized medicine,” “individualised medicine,” “personalized medicine,” “personalised medicine,” or “stratified medicine.” The right graph in Figure 1 shows how each of these group of publications appears in combination with the search terms “governance” and “policy.”

The results show that with respect to governance and policy the most frequent term authors refer to are related to personalized medicine, whereas the labels genetic medicine and personalized health are much less frequent. The use of the label personalized health is least frequent and increased during the last years (left Graph, Figure 2). The figure shows how frequently these labels appear in the Web of Science search in combination with the terms governance and policy. We re-ran the Web of Science search in Spring 2021 to include the entire year 2020.

The thematic analysis of the papers reveals four governance challenges for PH: (1) research infrastructure and practice (RP), (2) trust building (TB), (3) regulatory framework (RF), and (4) inclusion in health system (HS). An overview of the coding for each article can be found in the appendix to the paper (Table 1). The first governance challenge (RP) entails to create research infrastructure and practices that correspond to the new advances in genetic research but also to the new possibilities of data sharing and analysis that emerge from digitalization. The second challenge (RP) deals with the building of trust, notably among patients but also among citizens in general. The third governance challenge (RF) concerns regulation, for example regarding data storage and protection as well as non-discrimination of individuals based on their genetic profiles. Finally, the fourth governance challenge (HS) deals with the inclusion of PH into health systems, for example through the admission and reimbursement of new medications and treatments. The frequency of these categories in the main corpus of selected papers is depicted in the right side of Figure 2. The graph shows that research infrastructure and health system integration are discussed more frequently than trust building and regulation. Furthermore, the topic of health system integration appeared more often in recent years.

**TABLE 1:** Countries mentioned in author affiliations for different articles (main corpus).

Country	Mentioned in author affiliation or in the main text corpus
United States	18
United Kingdom	12
Canada	6
Italy	4
Netherlands	3
Australia	3
France	2
Germany	2
India	2
Turkey	2
Finland	2
Croatia	1
Malaysia	1
Thailand	1
Norway	1
Austria	1
South Korea	1
Denmark	1
Greece	1
Slovenia	1
Czechia	1
Kuwait	1
Switzerland	1
Belgium	1

**FIGURE 3:**
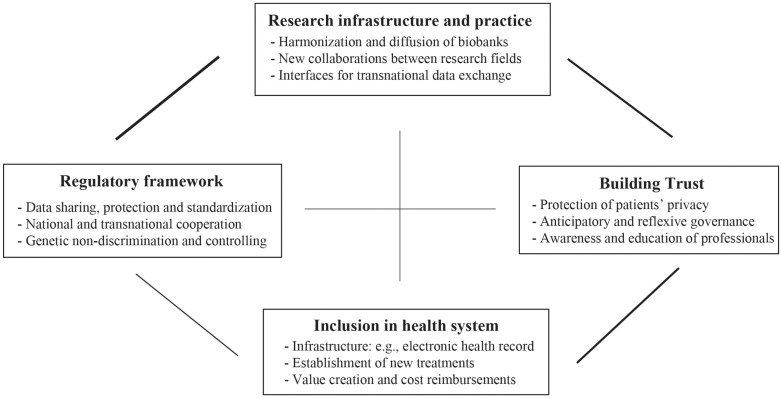
Four governance challenges.

Figure 3 illustrates the four governance challenges. Our thematic analysis reveals that the four identified challenges overlap, in terms of how they co-occur in different publications. The thicker the line between the different challenges, the more both governance challenges are discussed together within a publication (cf. Figure 2 based on data in Table 1). Concerning the links between the different challenges, we find that authors analyze most frequently the link between the governance of the research infrastructure and the regulatory framework. Further, studies examine the relation between trust building and inclusion in health systems on the one hand, and between trust building and research governance on the other. Researchers make only weak connections between research infrastructure and the inclusion of PH in health systems as well as between trust building and the regulatory framework (Figure 3).

The governance challenges relate to the policy problems that stakeholders (Kuhlmann, 2001; Ansell and Torfing, 2016) face in all health systems. Concerning PH, the most important stakeholders are: “promoters,” e.g., researchers, commercial and non-profit developers, sponsors of research and development, lobbyists and other advocates, “monitors,” e.g., editorial boards, regulatory bodies and curriculum committees, “providers,” e.g., clinicians and hospitals, and “users,” e.g., patient-based organizations (Juengst et al., 2012). These actors are similar to stakeholder constellations in other health policy issues (Lewis, 2006; Robert et al., 2017) and they will contribute in shaping policy responses to the governance challenges. At the same time, they will be affected by policies for PH. In the following sections, we discuss the four governance challenges in more detail.

### Research Infrastructure and Practice

The first governance challenge concerns the establishment and harmonization of infrastructures and practices that assemble the necessary information for research on prevention and treatment. Genetic data stored in biobanks is key to the development of PH services. Already for some time scholars have demanded to extend the creation of such infrastructure. A literature review dealing with the diffusion of biobank initiatives concerning PH holds: “Biobanking services must improve rapidly to serve the needs of personalized medicine and biospecimen research should be encouraged and supported at all levels from project funding to publication of results” (Hewitt, 2011, p. 112). Therefore, a very important element is that “formal governance structures are a common and necessary component of biobanks,” such as a formal access or oversight (Olson et al., 2014, p. 51) and ethics approvals are a common element (Zika et al., 2011, p. 100).

The creation of biobanks has advanced all around the world, in recent years (Kohane, 2011; Olson et al., 2014; Zawati et al., 2018), which offers new opportunities for scientific collaboration, such as in the Human Epigenome Consortium (Stunnenberg et al., 2016; Chiapperino and Panese, 2018). This development renders the harmonization of research infrastructures to allow for data exchange an even more important challenge (Stark et al., 2019). This is all the more important since the use of genomic data coincides with the new possibilities to analyze big data, for example algorithms (Vayena et al., 2018; Galetsi et al., 2019).

More specifically, harmonizing data sharing requires the creation of interfaces between research and health care applications, notably between different technological systems operating within and between organizations (Kawamoto et al., 2009; Heart et al., 2017). Particularly the establishment of compatible electronic health records (EHR) can create a database for research, linking genetic profiles and the history of health problems for patients (Kohane, 2011). EHR can become a “tool for genetic research, addressing concerns on accessibility, return of results and privacy and help in educate patients and healthcare providers” (Caenazzo et al., 2015, p. 4185). Such an infrastructure would allow to develop research possibilities even further, for example, in linking EHR and genetic data with geo-spatial data could provide new insights into individualized medicine (Schinasi et al., 2018).

Harmonization of research infrastructure is particularly difficult to achieve in decentralized contexts, such as in the United States (Joyner and Paneth, 2015, p. 999), but easier to achieve if there is a national health system, for example in the United Kingdom. It is likely to be even more difficult to facilitate research cooperation between nations, particularly regarding low- and middle income countries where resources for research are scarce. Nevertheless, the goal of harmonizing research infrastructures remains important, as cooperative efforts can contribute to dealing with important health problems, such as rare diseases (Boycott et al., 2017).

### Building Trust

The second challenge for governing PH is to build trust amongst citizens and patients against the background of technical innovations related to PH, especially when it comes to the use of their genetic and personal data (Platt and Kardia, 2015). This governance challenge refers to building trust between patients and practitioners, e.g., doctors and researchers. To build trust, researchers suggest to “(1) address the role of history and experience on trust, (2) engage concerns about potential group harm, (3) address cultural values and communication barriers, and (4) integrate patient values and expectations into oversight and governance structures” (Kraft et al., 2018, p. 3). Furthermore, researchers argue that there needs to be room for bottom-up developed rules and practices in the governance of biobanks, which includes citizens and helps to increase trust and transparency (e.g., Meslin, 2010). Scholars have used the terms reflexive and anticipatory governance to denote this particular governance challenge related to PH. Reflexive governance means that decisionmakers include citizens’ input in rule-making related to biobanks (e.g., Laurie, 2011). Anticipatory governance refers to the need to anticipate potential negative consequences of new technologies, such as loss of data in case of personalized medicine, when creating governance frameworks (Özdemir et al., 2011).

The literature points out that trust-building and the inclusion of citizens are important for governing PH to address the risk of discrimination based on genetic profiles (Feldman, 2012). Research based on interviews with geneticists, clinicians, computer scientists, ethicists, regulators, policymakers, and program administrators in the United States (US) involved in the creation of biobanks suggests that the collection of genetic data bears the risk that this data will eventually be used to interpret and frame health disparities by conflating race, ethnicity, and nationality with biological information (Lee, 2015). Consequently, scholars call for a solidarity-based approach to implement PH and medicine, i.e., governance practices need to ensure that discrimination can be avoided effectively (Prainsack, 2018). Thereby, one opportunity to create solidarity is to pursue data protection governance through community efforts (Wang et al., 2017).

Researchers have also pointed out that at the individual level, trust can be built by relying on health care professionals as intermediaries. One participant from a focus group research reported, “I might trust my doctor to use my information more than some third, fourth, fifth party removed in some library [biobank, precision medicine research program] somewhere. I know my doctor [.]” (Persaud and Bonham, 2018, p. 26). This quote illustrates the importance of the trust relationship between providers of health care services and patients when it comes to the sharing of individual health data (Laurie, 2011). Survey data confirms this insight: patients trust particularly their health care providers when it comes to sharing their personal data (Bühler et al., 2019). Against this background, the awareness and education of health care professionals into fostering patients’ trust into PH is crucial (Caenazzo et al., 2015).

### Regulatory Framework(s)

The third PH challenge concerns the establishment of regulatory frameworks. This governance challenges is related to the first one, which deals with the investment in the creation and harmonization of research infrastructures. Regulatory efforts in PH entail ensuring technical compatibility between different databases, such as biobanks, to encourage researchers and providers to collaborate in research for new treatments. In addition, regulatory efforts can work toward protecting citizens from discrimination and to ensure equitable access to the promises of personalized medicine.

Concerning data comparability, an important question for regulators is how to regulate data sharing between different stakeholders, notably patients, medical practitioners, hospital operators, pharma- and clinical researchers, as well as health insurers. Data sharing in PH does not only concern health systems within a country (Muddyman et al., 2013; Palanisamy and Thirunavukarasu, 2017), but also data exchange between different countries. This problem poses a challenge not only for high income countries but also for low- and middle income economies. A review of the biobanks in low- and middle-income countries demonstrates that there is a lack of harmonized data sharing systems and that data formatting is often not standardized (Zawati et al., 2018). Common regulations and standards can help to solve this problem. This regulatory dimension has a large transnational component and requires establishing regulations beyond single countries to support innovation. For example, pharmacogenomics requires the creation of a transnational regulatory regime that comprises a network including regulatory agencies, academic scientists and industry, and aim at creating a space for data sharing and to set standards that span across jurisdictional boundaries (Hogarth, 2012).

Another important theme for regulation of PH is to ensure genetic non-discrimination of individuals through the information contained in biobanks or EHR in order to protect privacy concerns (Williams et al., 2016). This seems somewhat self-evident and necessary. Nevertheless, researchers have held that it might be a challenge to implement such regulations as they could slow down innovation (Juengst et al., 2012). For example, the United States Congress passed the Genetic Information Non-discrimination Act (GINA) in 2008 (Dressler and Terry, 2009; Feldman, 2012; Feldman and Darnel, 2013; Green et al., 2015; Rothstein, 2018). The legislation aims to rule out genetic discrimination regarding health insurance admission and employment. Feldman (2012), p. 743 states that “GINA prohibits insurers from using genetic information to adjust group or individual premiums, deny coverage, or impose preexisting condition exclusions, and makes it illegal for them to require or request genetic testing or intentionally obtain genetic information.” The law received overwhelming support in the United States Congress and has important implications for medical providers and health care organizations, which must familiarize themselves with the specificities of the act. Other countries, for example European Union (EU) member states, have also legislated to prevent discrimination on a genetic basis (Borry et al., 2012). Protection against discrimination and diffusion of health care innovation is a transnational regulatory challenge, cf. the jurisdiction of European directives (Salas-Vega et al., 2015). The EU’s general data protection regulation and the Organization for Economic Co-operation and Development’s recommendations for digital health governance are two cases that exemplify the transnational dimension (Vayena et al., 2018).

In addition to rulemaking, rule implementation is an important challenge. In other words, data regulation is more than passing laws, it also concerns the implementation of such regulations in practice. For example, scholars have emphasized the need for data controllers who are able to support researchers in dealing with legal challenges: “It cannot be the responsibility of the researcher who wants to access data to handle the legal intricacies of EU and national data protection legislations; this must be done by the data provider who acts as a data controller” (Kuchinke et al., 2016, p. 17). Such practices not only protect researchers from legal challenges emerging from research dealing with PH, but they also make the research process more transparent (Kaye et al., 2018), and therefore increase the trust in PH and medical innovation. Eventually, the regulatory architecture for PH needs to include ethics regulation and a committee for genomic research to ensure “accessibility, return of results and privacy and help in educate patients and healthcare providers” (Caenazzo et al., 2015, p. 4185).

### Integration Into Health Systems

The fourth governance challenge is to integrate PH (Hedgecoe, 2004; Nwaru et al., 2017; Minari et al., 2018) into existing health systems, in other words, to integrate PH in the regulation, financing, and provision of public health and health care (Trein, 2018). In many countries, for example in Japan, Great Britain, and the United States, policymakers have included precision medicine schemes in the context of national health systems (Minari et al., 2018).

But how exactly could PH be integrated into existing health systems? One proposition in the literature suggests to assess the inclusion of PH in national health systems along six key themes: healthcare system, governance, access, awareness, implementation, and data. Specifically, the governance dimension, should entail a national strategy, comprehensive legislation and guidelines, as well as an ethical, social, and legal framework regarding the provision of personalized medicine and genetic data. Further indicators are a national research center or large-scale research initiative, a consumer test legislation or code of conduct, and working groups with multiple stakeholders (Chong et al., 2018, p. 2 and Table 2). For example, the United States created the national Precision Medicine Initiative (PMI). Its goal is to create more genetic research programs, which should ideally result in better health care programs (Sabatello and Appelbaum, 2017). Scholars have linked the PMI to the idea of a genetic citizenship, which entails the exchange of personal information in exchange for information from genetic research to make the best health-related choices for themselves. Put differently, this concept entails a new contract between citizens and the state – respectively, health care providers – and entails risks, benefits, and responsibilities for each participant (Sabatello and Appelbaum, 2017).

**TABLE 2:** Synopsis of reviewed papers (Main corpus).

Reference	Region	Methodology	Key contents and main results	RP	TB	RF	HS
Dry et al. (2017)	United States (California)	Deliberative community engagement (*N* = 51)	Biobank governance and oversight recommendations Educate the public, share samples broadly, monitor researcher behavior Use informative consent procedures and involve community members	✓	✓	✓	
Caenazzo et al. (2015)	*No empirics*	Commentary	Pairing disease biobanks with electronic health records (EHR) for research Specific ethics committees for each biobank to improve governance Committees to set up EHR utilization guidelines and to address concerns	✓	✓		
Hewitt (2011)	*No empirics*	Literature review	Description of advances in biobanking and biospecimen research Quality management and professional organization of biobanks Improvement of collection models and patient protection	✓	✓		
Meslin (2010)	United States (Indiana)	Case reviews (*N* = 279 and 1000)	Building trust and transparency in biobanks governance structures Top-down steering to be complemented with bottom-up governance Bottom-up strategies to include researchers and local communities	✓	✓		
Zawati et al. (2018)	Low/middle income countries	Literature review/meeting notes	Review of challenges and opportunities identified by biobank researchers Informed consent, access policy, and data sharing is critical Biobanking should account for political and social conditions	✓	✓		
Chalmers et al. (2016)	Australia, Germany, Japan, Singapore, Taiwan, United Kingdom, United States	Country review (7 countries)	Operational, sustainability, and funding challenges in biobanking Resources, viability, and usefulness of running biobanks Technologies and strategies to minimize overly complex structures	✓		✓	
Kaye et al. (2018)	*No empirics*	Commentary	National governance hinders international exchange of research data Need of public consultations on access and use data-sharing issues Digital technologies to encourage accessibility, transparency, accountability	✓		✓	
Laurie (2011)	United Kingdom	Conceptual investigation	Reflexive governance as approach without specific basis in law High-level policy documents guide decisions and practice Commitment among participants, researchers and society avoids regulation	✓		✓	
Lee (2015)	*No empirics*	Ethnographic research	Institutional practices of classifying and creating taxonomies Biobanks as political artifacts framing health differences in populations Avoid conflation of race, ethnicity, and nationality with biological differences	✓		✓	
Sardas and Kendirci (2019)	*No empirics*	Commentary	Systems approach to pharmacovigilance and risk governance Need for centers for panvigilance and global clinical trials Harmonization of biomakers for product development and trials	✓		✓	
Chan and Erikainen (2018)	United States	Commentary	Term “precision medicine” is overly ambitious Systems approaches relate to multiple ideas and aims United States genetic research is no public good due to the private health care providers	✓			✓
Kohane (2011)	*No empirics*	Review article	Use of electronic health records and related information for genetic research Link genetic studies to clinical health care delivery Such research can be conducted while maintaining patient privacy	✓			✓
Andreu-Perez et al. (2015)	*No empirics*	Statistics (2007–2014), case reviews	Development of big data in biomedical and health informatics Big data will advance disease management (diagnosis, prevention, treatment) Challenges in privacy, security, data ownership/stewardship, governance	✓			
Boeckhout and Douglas (2015)	Netherlands	Case study	Biobanking infrastructures positioned between healthcare and research Changing relationship between care and research biobank governance Medical responsibilities on both sides require new forms of governance	✓			
Hawkins and O’Doherty (2011)	Canada	Semi-structured interviews	Microbiome research adds to biobanking and data sharing complications Revisit of privacy, consent, ownership, results, governance, and benefit sharing Minority views and facilitation to be considered in governance	✓			
Heeney and Kerr (2017)	United Kingdom (Scotland)	Literature review	Standardization of data sharing and access in biobanking Data access governance needs to be flexible and reflexive Wider data sharing environment and local specificities need to be included	✓			
Olson et al. (2014)	*No empirics*	Literature review	Support of clinical genetics by carefully designed biobanks Setting up a biobank and linkage to electronic health records Recruitment, investigations, re-use of data, and sustainability	✓			
Özdemir et al. (2017)	*No empirics*	Conceptual article	Technology foresight analysis defining “environtome” and “social proteome” Personalized health beyond genomics with big data technologies (proteomics) Synergistic value of social and biological proteomes in psychology	✓			
Palanisamy and Thirunavukarasu (2017)	*No empirics*	Literature review	Success of big data healthcare applications depends on architecture and tools Diversified data analytical capabilities for handling sources of data needed Eco systems to include patients, doctors, hospitals, researchers, and insurers	✓			
Ingrams (2019)	*No empirics*	Conceptual article	Application of Dahl’s theory of democracy to health data governance Recognition of the role of citizens in policy making Discussion of control and autonomy of citizens on health data		✓	✓	✓
Minari et al. (2018)	Japan, United Kingdom, United States	Commentary	Ethical dimensions in national strategies for precision medicine Mitigate undesirable impact on privacy, commercialization and public trust Approaches to consider equity, social justice, resources and politics		✓	✓	✓
Dove (2015)	*No empirics*	Literature review	Review of international disease and database consortia and projects Coordination is critical in international governance of biobanking Privacy laws need to be harmonized to allow for data sharing		✓	✓	
Persaud and Bonham (2018)	*No empirics*	Commentary	Crucial role of health care providers in creating trust of patients Physicians to serve as agents and mediators in precision medicine programs Patients tend to trust doctors the most with their genetic data	✓			✓
Sabatello and Appelbaum (2017)	United States	Commentary	Active, informed participation in research through “genomic citizenship” Analysis of risks and benefits for participating in such initiatives Individual empowerment as a result of genetic testing to remain doubtful	✓			✓
Woolley et al. (2016)	United Kingdom, United States	Case studies	Translational biomedical research requires large pools (“citizen science”) Initiatives to include an analysis of the role of citizenry Terms “participation,” “involvement,” and “engagement” to be clarified	✓			✓
De Vries et al. (2019)	United States (Michigan)	Democratic deliberations (*N* = 180)	Moral concerns of donors in biobanking Participants worry about ethical problems of consent Public trust and (dis)trust in science to be addressed		✓		
Kraft et al. (2018)	United States (Greater San Francisco Bay area)	Focus groups (*N* = 122)	Build/maintain long-term, trust beyond consent with patient-participants Address experience, concerns, cultural values, communication barriers Integrate patient values to enhance trustworthiness		✓		
Platt and Kardia (2015)	United States	Survey of citizens (*N* = 447)	Public trust in health information sharing systems Primary care provider and psychosocial factors positively influence trust Privacy concerns and knowledge about sharing are negatively associated		✓		
Rose (2013)	United States	Case study	Trust in Patient Advocacy Organizations (PAO) Limited industry funding to promote PAO trustworthiness Separate fundraising and policymaking, increased transparency		✓		
Doheny et al. (2018)	United Kingdom (England, Wales)	Semi-structured interviews (*N* = 34)	Patients to receive an updated interpretation of their genetic information System responsibilities come with governance and legal issues Interplay with professional obligations (duties, responsibilities, obligations)			✓	✓
Hedgecoe (2004)	United Kingdom, United States	Semi-structured interviews	Impact of pharmacogenetics technology on clinical practice Industry and researchers have a simplified view compared to clinicians Clinical context is widely resistant to the revolution from pharmacogenetics			✓	✓
Kirchhof et al. (2018)	Germany	Case study	Stratified prevention as a major change for health policy in Germany Individual control and understanding of health information required Updated governance and evidence-based development of taxonomies			✓	✓
Prainsack (2018)	*No empirics*	Conceptual article	Solidarity concepts to depart from the assumption of rational individuals Relational understanding of personal and collective preferences Policies and practices to focus on the overlap of the two			✓	✓
Kuchinke et al. (2016)	*No empirics*	Legal analysis	Development of a legal assessment tool for data access and sharing Provides assessments and recommendations for researchers Model develops usage scenarios and requirement clusters for data sharing			✓	
Hogarth (2012)	European Union, United States	Desk research, interviews	Pharmacogenomics to transform drug discovery and development Transnational regulatory regime encompassing national actors needed Harmonization and standards setting across jurisdictional boundaries.			✓	
Muddyman et al. (2013)	United Kingdom	Case study	Analysis of a data management system implementation (UK10K) Reconciliation of data-sharing principles and system practicalities Three key issues: study recruitment, data release and data access			✓	
Tupasela and Liede (2016)	Finland	Desk research, interviews	Management of data in biobanks and sharing infrastructures Practical implementation of the European Data Protection Directive Governance to consider privacy concerns over individual data			✓	
Song et al. (2017)	*No empirics*	Topic model	Patent landscape dominated by therapeutic patents Focus on areas of oncology and neurodegenerative and infectious diseases Insights for future technology planning			✓	
Boccia et al. (2017)	Italy	Commentary	Integration of genomics into National Health Service Areas of focus: prevention, diagnosis, and care Consider effectiveness (evidence-based) and sustainability (cost-effectiveness)				✓
Bowman et al. (2016)	United States (Arizona)	Commentary	Availability of health and blood tests in local pharmacies Interpretation of health data shifts from professionals to consumers Individuals circumventing physicians entails policy concerns and safety risks				✓
Chong et al. (2018)	Indonesia, Malaysia, Singapore, Thailand	Scoping review, semi-structured interviews (*N* = 11)	Adoption of personalized medicine in Southeast Asia’s health systems Governance, access, awareness, implementation, and financing are reviewed Balancing equity among populations and improving efficiency are critical				✓
Feldman (2012)	United States	Commentary	Genetic Information Non-discrimination Act in health insurance/employment Examination of fear and reality of genetic discrimination Medical providers must be familiar with the terms of the law				✓
Geller et al. (2014)	*No empirics*	Commentary	Ethical, legal and social implications of genetic research on public health Infectious disease management procedures in health care practice are affected Balance health-related benefits/harms with impact of policy interventions				✓
Gray et al. (2019)	*No empirics*	Literature review	Precision medicine to add value in lifestyle medicine beyond genomics Provide suitable types of support to people to adopt a healthy lifestyle Holistic and person-centered approach to be chosen over a mere technological				✓
Özdemir et al. (2011)	*No empirics*	Conceptual article	Anticipatory governance for vaccinomics and post-genomic technologies Response to impredictability of consequences of a technology in early stages Anticipation with participatory foresight to respond to inherent uncertainties				✓
Ricciardi and Boccia (2017)	*No empirics*	Commentary	Citizen engagement as prerequisite for policy change in public health Governance, consent, trust, data-knowledge cycle to be improved Adopt/adapt technology assessment while retaining humanity/community				✓
Vozikis et al. (2016)	European Union (member states)	Conceptual article	Pricing and reimbursement policies of genomic tests Strategies to include universal access, cost monitoring and appropriate use Develop research capacity and invest in human resources				✓

*RP, Research infrastructure and practice; TB, Trust building; RF, Regulatory framework; HS, Inclusion in health system.*

In addition, including PH into existing health systems requires to fit new services and practices with national regulations and financing schemes. In the following, we illustrate this problem based on five examples.

1. Firstly, this entails the implementation of EHR in existing routines of health care and to include information beyond the clinical health data (Heart et al., 2017; Lu et al., 2018).
2. Secondly, it requires the assessment and certification of genetic tests’ actual public health value. Nowadays, consumers can choose between a widening array of genetic tests but it is not clear to what extent these tests effectively contribute to improving individual and public health and should therefore be reimbursed by health insurance (Hall et al., 2010; Caulfield and McGuire, 2012).
3. Thirdly, there is the challenge to approve new treatments (Bertier et al., 2016), for example orphan drugs or personalized drugs (Garrison et al., 2008) as well as new cancer therapies, such as precision immunotherapy for metastatic melanoma (Chin-Yee et al., 2018, p. 383).
4. The fourth element about integration is the reimbursement of these new treatments and their inclusion in health care payers’ plans (Meckley and Neumann, 2010; Messner et al., 2016). New personalized treatments and drugs tend to be expensive (Degtiar, 2017), which raises the question how to ensure equity in access (Williams et al., 2016).
5. Fifth, the literature points out that PH will increase or reduce the disparities between medical health care and public health. On the one hand, genetic testing provides new possibilities for preventative medicine, such as stillbirth prevention (Ker, 2018). On the other, the predictive power (Bourret et al., 2011; Juengst et al., 2012) of precision medicine might re-enforce health inequalities rather than decreasing them (Khoury and Galea, 2016; Chin-Yee et al., 2018). The last point is particularly relevant since it is very unlikely that “personalized” medicine with individually designed plans for prevention and treatments becoming reality in the near future. The development of stratified medicine that considers genetic variations between different groups is more likely. This makes equity problems all the more relevant (Juengst et al., 2012; Tutton, 2012; Minari et al., 2018).

At an organizational level, these policy challenges need to be absorbed by the existing structure of the national health systems. For example, in Italy, “the State-Region conference approved and published the national plan of public health genomics. A further step has recently been made with the approval of a ‘National Plan for Innovation of the Health System based on omics sciences.”’ This plan includes measures to introduce the use of big data in the health system, but it also aims to support economic growth through investment in PH (Boccia et al., 2017, p. e12782-2). Taking a comparative perspective, scholars have focused on organizations that are responsible for managing genetic tests. In the United States, the Centers for Medicare and Medicaid Services and other government agencies are responsible for genomic testing and for creating regulatory standards to integrate genomic testing in clinical practice. The Secretary’s Advisory Committee on Genetics, Health and Society and the National Human Genome Research Institute deal with the question on how genetic tests can be reimbursed for patients (Vozikis et al., 2016, p. 353). In the EU, genetic tests are regulated by the EU directive on medical device regulations which requires certification of the device to have a “*Conformité Européenne*” (CE) mark. Reimbursement of tests is regulated differently in each EU country. For example, “in Germany, it is administered by *Der Gemeinsame Bundesausschuss* (GBA), in France by *La Haute Autorité de Santé* (HAS), in the United Kingdom by the *National Health Service* (NHS), in Italy by *Il Servizio Sanitario Nazionale* (SSN), and in Spain by *El Instituto Nacional de la Salud (INS)*” (Vozikis et al., 2016, p. 354).

Up to know, it remains open how different political actors have taken action to integrate personalized health into the contexts of different health systems. Nevertheless, a comprehensive analysis on how PH has been included in health governance is lacking for European countries. What is more, we know much less about the role of health system integration of PH in low- and middle-income countries.

## Conclusion

This article presents the results of a scoping review of the literature on PH governance. Our review shows that policymakers face particularly four governance challenges when putting PH into practice: (1) creating, maintaining and harmonizing an infrastructure for research, (2) building and fostering trust in PH amongst citizens in general and patients in particular, (3) establishing regulatory frameworks to ensure cooperation and to avoid discrimination, and (4) integrating PH into existing health systems.

These four challenges are relevant for practitioners and researchers alike. Concerning the research agenda that lies ahead, our review suggests that scholarship dealing with the implementation of PH should focus on research questions emerging from the four challenges for governing personalized health. Scholars aiming at a specific research question could direct their efforts at the link between trust building and regulatory frameworks on the one hand, as well as on the relationship between the research infrastructures and health system integration on the other. Both topics are highly important for realizing PH in practice. Nevertheless, according to our review, scholars have so far devoted limited attention to these questions. Furthermore, there is room for a rigorous empirical comparison that analyzes how governments in different countries with different health systems have addressed the four governance challenges outlined above. Specifically, researchers should explore why there are (potential) differences and similarities in how governments address the four challenges, for example in taking into account recent developments in research on health care systems (e.g., Papanicolas et al., 2013; Reibling et al., 2019).

The four governance challenges uncovered by this literature review refer to other topics, which scholars of governance raised in the literature, such as transparency, accountability, protection of human rights, international standards, and citizen participation (Papadopoulos, 2007; Merry, 2011; Ansell and Torfing, 2016). Our findings show that the demand for trust building requires a transparent usage of genetic data. Furthermore, trust building entails that citizens can participate actively in the research process. We also demonstrate that creation of a regulatory framework and standards is part of the literature personalized health governance, which is important to ensure human rights protection at the national and a global level. Furthermore, the development of regulations and standards is relevant to govern risks, determine liability and to protect individuals from unequal treatment regarding personal health. Thus, our review paves the way for future research on the governance of personalized health.

In addition, the four governance challenges are relevant for practitioners as they give an overview of the most relevant policy challenges for PH practice. To be clear, the four challenges that arise from our scoping review are an empirical mapping of the state of the literature, rather than a normative agenda. We do not call for a top-down approach to addressing these challenges by way of central state intervention. According to our interpretation, it must be the goal of PH policymaking to address these four challenges in a balanced manner. To achieve this aim, PH governance requires including a variety of stakeholders in order to co-produce sustainable governance arrangements, rather than to govern in a hierarchical fashion from the top. We are well aware that such demands are ubiquitous and relevant for many policy problems. Nevertheless, our results show, however, that this approach is also relevant for the field of personalized health.

Finally, we would like to point the reader to the limitations of this review. Firstly, our search is based on one database (Web of Science). This database has a broad reach and is likely to pick up published and gray literature. The search retrieved documents classified as Articles, Editorial Materials, Proceedings Papers, Letters, Reviews, Book Chapters, Meeting Chapters, Books, and Book Reviews. Thus published research that is only indexed in other databases might have been missed. Secondly, our search did not cover press articles. Future research could reveal how the scientific debate on personalized health relates to the debate in newspapers.

## Author Contributions

All authors listed have made a substantial, direct and intellectual contribution to the work, and approved it for publication.

## Conflict of Interest

The authors declare that the research was conducted in the absence of any commercial or financial relationships that could be construed as a potential conflict of interest.
